# Microarray-based component-resolved diagnosis of latex allergy: isolated IgE-mediated sensitization to latexprofilin Hev b8 may act as confounder

**DOI:** 10.1186/2045-7022-3-11

**Published:** 2013-03-28

**Authors:** Sarah Schuler, Giovanni Ferrari, Peter Schmid-Grendelmeier, Thomas Harr

**Affiliations:** 1 Allergy Unit, Department of Dermatology, University Hospital of Zurich, Zürich, Switzerland; 2 Servizio di allergologia e immunologia clinica, Ospedali Regionali di Bellinzona e di Lugano, Bellinzona, Switzerland; 3 Service d’immunologie et d’allergologie, Unité d’allergologie, Hôpitaux Universitaires, Geneva, Switzerland

**Keywords:** Component-resolved diagnosis (CRD), Carbohydrate cross-reactive determinants, Hev b 8, Latex, Profilin

## Abstract

Immediate type allergy to latex is still a widespread problem. Latex-allergic patients undergoing diagnostic and operative medical procedures are at risk of potentially life-threatening reactions. Accurate diagnostic methods are therefore crucial. The aim of this retrospective study was to discriminate between sensitization and relevant allergy to latex based on an easy and suitable diagnostic approach. In 14 patients with clinical symptoms and 27 controls, latex skin prick tests (SPT), IgE against latex (CAP) and serological component resolved specific latex-allergen determination (Hev b1, b3, b5, b6, b7, b8, b9, b10, b11) based on ImmunoCAP ISAC were performed. SPT correlated very well with clinically manifest latex-allergy demonstrating a high specificity (95%) (and a low sensitivity). However, CAP levels to crude latex could not safely discriminate between purely sensitized and latex-allergic patients. The majority of patients mono-sensitized to the latex profilin Hev b8 did not suffer from any relevant symptoms upon contact with latex. However, in two patients with latex-allergy diagnosed by elevated specific IgE only sensitized against Hev b8, additional sensitization to carbohydrate cross-reactive determinants (CCD) was found. In the case of positive serum IgE against latex and negative SPT, component-resolved diagnosis including IgE against specific latex-proteins, specially Hev b8, and carbohydrate cross-reactive determinants (CCD) is a useful tool to discriminate between latex-sensitization and latex-allergy.

## 

Natural rubber latex (NRL) contains a variety of proteins. Thirteen latex-proteins have been identified and characterized [1]. Hev b5 and Hev b6 play a major role in sensitized healthcare-workers [2,3] and in patients with respiratory symptoms [4]. Healthcare-workers seem having persistent skin-test-reactivity despite avoiding latex-contact [5]. Spina bifida patients typically show sensitizations to Hev b1 and Hev b3, but - to lower extent - also to Hev b5 and Hev b6 [6] and Hev b7 [7]. Hev b8 is a profilin, involved in certain latex-fruit-syndromes [8]. Hev b9, an enolase [9], was described in few cases of latex-sensitization in healthcare-workers but not in spina bifida [10]. Another risk-group for potential serious latex-associated side-effects are obstetric patients [11].

Correct identification of latex-sensitized patients with true latex-allergy and increased risk for potentially severe reactions during medical procedures is a major task. In case of unknown clinical history, conventionally determined latex IgEs is insufficient for discrimination between latex-sensitization and latex-allergy. Therefore, complete avoidance of latex-containing materials is recommended in every patient with positive IgE to latex whether sensitized or allergic. However, avoidance of latex-devices may cause logistical problems and higher healthcare-costs. Newer approaches include use of component-resolved diagnosis by direct determination of specific IgE to allergen-subgroups [12,13] like latex-allergens as well as indirect determination of latex-allergens by basophil activation tests [14]. As healthcare-funding is under pressure today, the aim is to filter patients at risk by a simple cost-effective diagnostic strategy.

Besides protein allergens, specific IgE antibodies to carbohydrates are under debate in certain cases of allergic reactions. These carbohydrate cross-reactive determinants (CCD) are mainly detected by specific antibodies to pineapple glycoprotein bromelain. It is debated if specific IgE to CCD play a role in latex-allergy and if bromelain is sufficient to detect all CCD-positive patients [15,16].

## Findings

### Patients, materials and methods

Data-analysis was approved by local ethics-committee (2009–0508). A total of 41 patients with elevated specific latex IgE (ISAC: 41 patients, ImmunoCAP IgE: 39 patients, Prick: 33 patients) were recruited from our Allergy Unit and analysed retrospectively. A detailed medical history with special regard to possible latex-associated symptoms upon direct contact was recorded for every patient.

Specific IgEs to latex were detected by Phadia ImmunoCAP (allergen code k72 Latex, Hevea brasiliensis). IgE level of 0.35 kU/l was defined as cut-off; values above 0,35 kU/l were considered positive.

In the ImmunoCAP ISAC-Microarray specific IgE to a broad variety of allergens was measured using biochip-technology, according to the manufacturer (Phadia). Allergen-specific IgE-antibodies were detected by fluorescence-labeled anti-IgE antibodies with laser-scanner, and analysed by Phadia MIA software.

Among analysed allergens (all but two recombinant) the following were of particular interest:

Latex (rHev b1, rHev b3, rHev b5, rHev 6.01, rHev 7, rHev b9, rHev b10, rHev b11), nMUXF3 (sugar-epitope from Bromelain, CCD-marker) and profilins (rHev b8 [Latex, Hevea brasiliensis], rPhl p12 [Timothy grass, Phleum pratense], rBet v2 [Birch, Betula verrucosa], rMer a1 [Annual mercury, Mercurialis annualis], nOle e2 [Olive, Olea europea]).

31 patients were analysed with following latex-allergen panel: Hev b1, b3, b5, b6, b8. 10 patients had additional analyses of the following latex-allergens: Hev b7, b9, b10, b11.

Latex skin prick tests (SPT) were performed with Alyostal 903 Latex (Stallergènes, total latex-protein content: 20 microgram/ml (100 I.R./ml)). Single protein-concentrations: 0.15 microgram/ml for Hev b1, 0.23 microgram/ml for Hev b3, 0.46 microgram/ml for Hev b5 and 4.53 microgram/ml for Hev b6.

Statistical calculation was done using unpaired t-test to evaluate significant difference between both groups (level of IgE in latex-allergic versus sensitized only persons), expressed by p-value.

## Results

**Figure 1:**
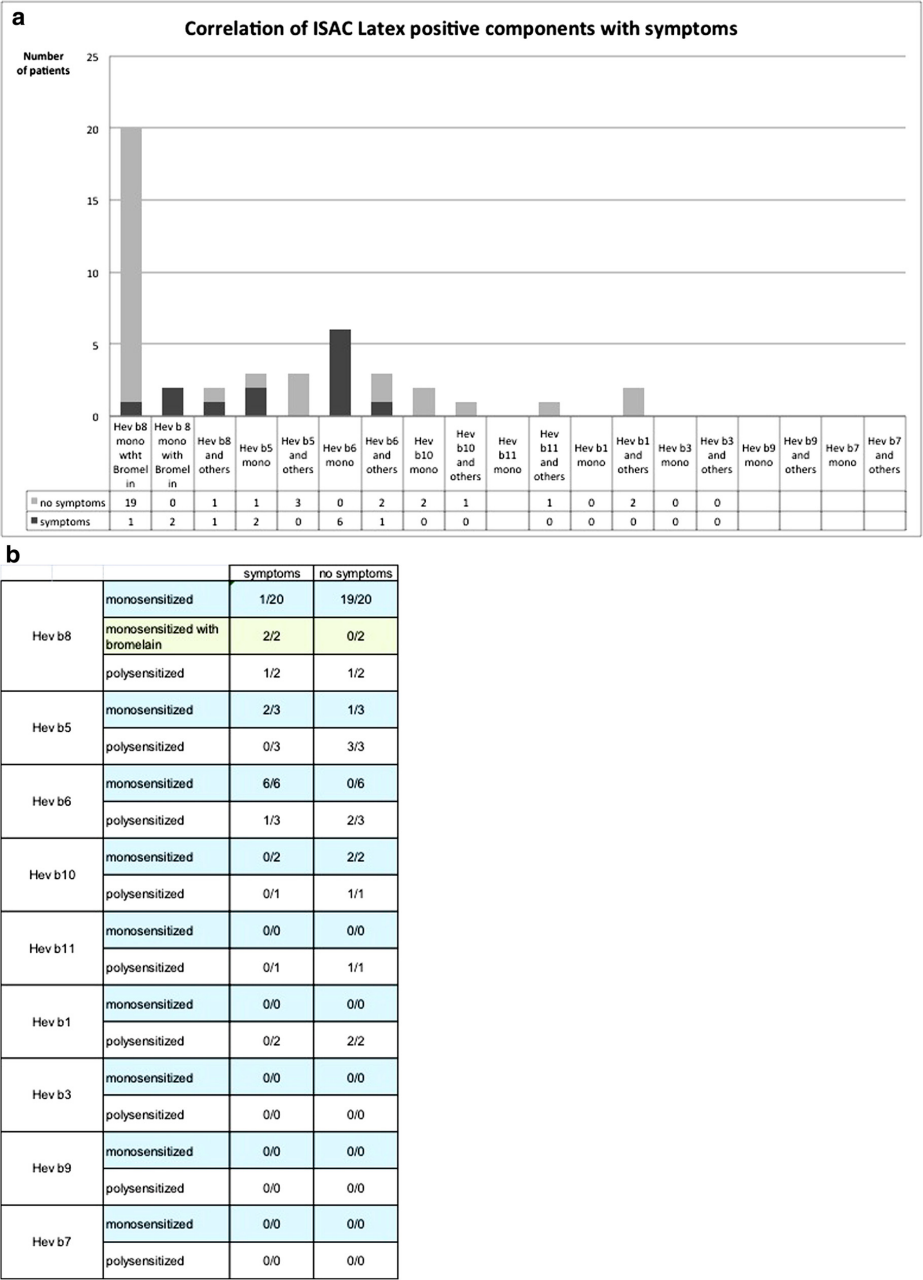
(a) Sensitization profile to different latex proteins with respect to symptoms (b) Symptoms in relation to sensitization to selected latex proteins.

Out of 41 patients with latex-sensitization as demonstrated by IgE (ImmunoCAP and/or ISAC, 26f/15 m, average age 38 years) only 14 patients reported relevant clinical symptoms upon direct contact with latex and where therefore considered as latex-allergic (Table 1). 37/41 patients showed at least one sensitization to a recombinant latex-allergen by ISAC. Two patients with relevant and two patients with latent sensitization to latex detected by ImmunoCAP were completely negative to available recombinant latex-allergens (Patients 13/14 and 38/39, Table 1). 20 patients were mono-sensitized to Hev b8. Only one of these showed symptoms upon latex-contact. Two patients had additional specific antibodies to bromelain (CCD), and were considered allergic to latex according clinical history. Only two patients with elevated IgE to Hev b8 were also sensitized against other latex-allergens. Hev b6 mono-sensitized patients showed clinical symptoms upon latex-contact. Only two out of six patients sensitized to Hev b5 showed clinical symptoms, independently of mono- or poly-sensitization, (Figure 1).

**Table 1:** Patients characteristics

**Patient no.**	**Age**	**Gender**	**Reported clinical symptoms upon direct latex exposure**	**Sensitization profile to skin prick test**	**Sensitization profile to ImmunoCAP [kU/l]**	**Sensitization profile to latex components (Hevein b)**	**IgE to bromelain (CCD)**
			**(Potential cross reactive symptoms to food not included)**				
1	36	M	intraoperative anaphylaxis	1	12.1 (3)	6	
2	38	F	Contact pruritus, dyspnea	1	5.64 (3)	6	
3	31	F	pruritus, urticaria, angioedema	2	3.27 (2)	6	
4	23	F	contact pruritus	1	7.58 (3)	6	
5	60	F	contact pruritus, nausea, sweating	3	8.1 (3)	6	
6	73	F	contact pruritus	no data	39.8 (4)	6,8	
7	58	F	anaphylaxis upon initiation of anesthesia	0	2.14 (2)	6	
8	29	F	contact pruritus	no data	85.1 (5)	8 (0.7)	Positive (7.8)
9	16	M	contact pruritus,	0	5.42 (3)	8 (7.6)	Positive (3.0)
10	44	F	contact pruritus	no data	1.56 (2)	8	
11	21	F	contact urticaria	3	10.3 (3)	5	
12	60	F	contact pruritus	0	no data	5	
13	40	F	latex allergy not other specified	1	2.19 (2)	negative	
14	27	F	contact urticaria	0	0.93 (2)	negative	
15	50	F	no known latex allergy	no data	0.06 (0)	1,5,6	
16	26	F	no known latex allergy	0	0.06 (0)	1,5,6,10,11	
17	83	F	no known latex allergy	no data	0.06 (0)	5,8	
18	62	M	no known latex allergy	0	0.05 (0)	8	
19	24	M	no known latex allergy	0	9.59 (3)	8	
20	21	F	no known latex allergy	no data	4.53 (3)	8	
21	55	F	no known latex allergy in the documentation	no data	2.53 (2)	8	
22	25	M	no known latex allergy	no data	1.02 (2)	8	
23	30	M	no known latex allergy	0	21 (4)	8	
24	32	M	no known latex allergy	0	no data	8	
25	15	M	no known latex allergy	0	3.73 (4)	8	
26	25	M	no known latex allergy	0	0.79 (2)	8	
27	42	F	no known latex allergy	0	1.64 (2)	8	
28	40	M	no known latex allergy	0	0.94 (2)	8	
29	20	F	no known latex allergy	0	1.03 (2)	8	
30	35	F	no known latex allergy	0	1.38 (2)	8	
31	64	F	no known latex allergy	0	0.37 (1)	8	
32	22	M	no known latex allergy	0	0.62 (1)	8	
33	22	F	no known latex allergy	0	0.81 (2)	8	
34	29	M	no known latex allergy	0	1.3 (2)	8	
35	27	M	no known latex allergy	0	0.35 (1)	8	
36	91	F	no known latex allergy	0	0.34 (0)	8	
37	39	F	no known latex allergy	1	2.59 (2)	5	
38	25	M	no known latex allergy	0	1.13 (2)	negative	
39	40	M	no known latex allergy	0	1.79 (2)	negative	
40	18	M	no known latex allergy	0	0.06 (0)	10	
41	51	F	no known latex allergy	0	0.06 (0)	10	

Two patients with contact urticaria/pruritus to latex were identified as anti-CCD positive with exclusive sensitization to Hev b8 (Figure 1). Otherwise, anti-CCD antibodies were identified neither in allergic nor in latex-sensitized patients.

**Figure 2:**
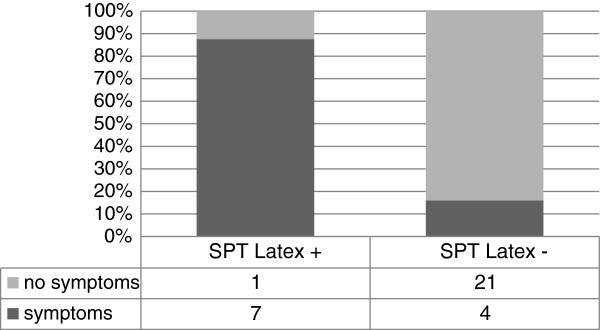
Correlation of latex sensitization identified by skin prick test and clinical symptoms.

Thirty-three SPTs were performed in 41 patients. Eight positive latex SPTs were found. 7/8 SPT positive patients were considered as clinically relevant, with allergic symptoms upon latex-contact. In 4/25 cases latex SPT was false-negative, as identified by ImmunoCAP or ISAC. Sensitivity of latex SPT was only 33% whereas specificity was 95% (Figure 2).

**Figure 3:**
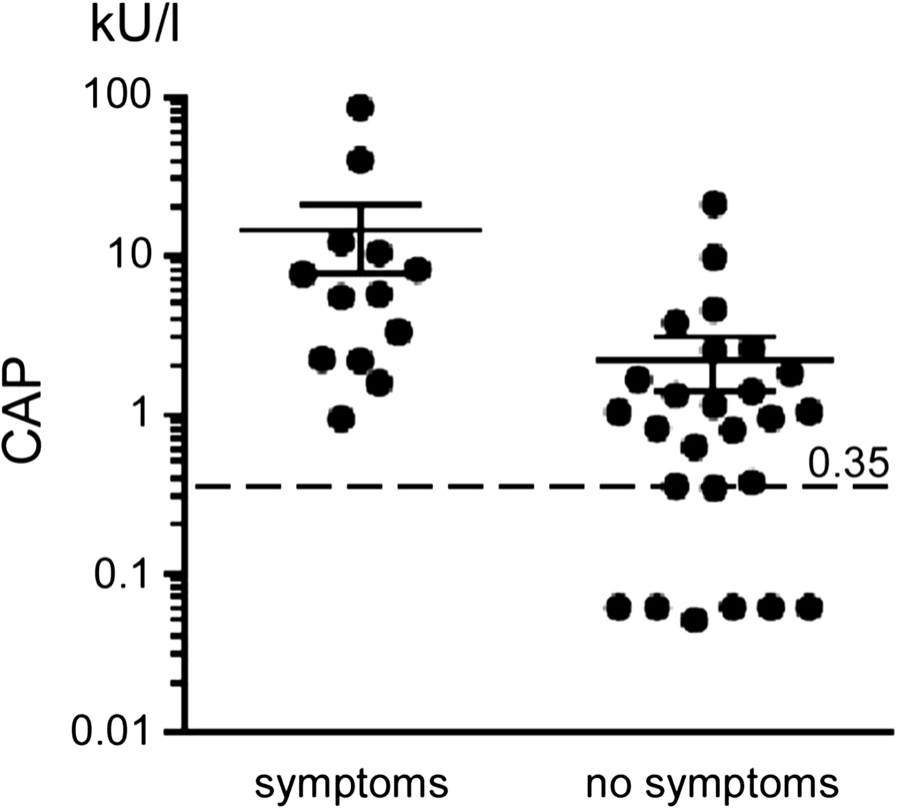
Latex CAP IgE levels in latex allergic (with symptoms with latex contact) versus only latex-sensitized patients (with no symptoms).

Patients with symptoms had significantly higher specific IgE latex CAP compared to latex-only sensitized individuals (Figure 3, p < 0.01).

**Figure 4:**
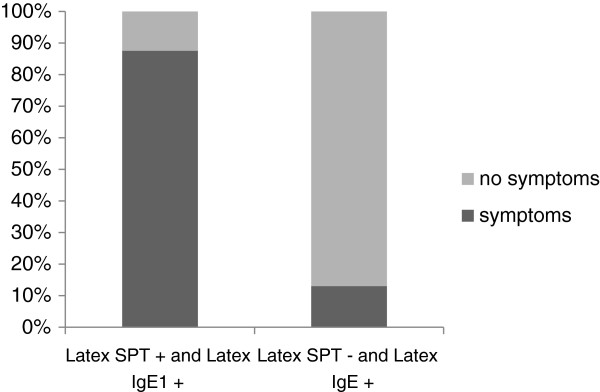
Clinical significance of combined use of latex skin prick test and specific IgE to latex.

Patients that were double-positive in the SPT and IgE latex CAP showed clinically relevant symptoms in more than 75% of cases, whereas SPT negative and latex IgE CAP positive patients had clinically relevant sensitization in only 13% (Figure 4).

**Figure 5:**
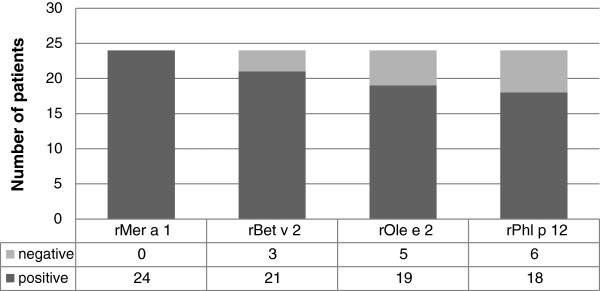
Cross-sensitivity profile to different profilins in Hev b8 positive patients.

Hev b8 showed a high concordance to other profilins. The highest correlation was identified to Mer a1 (Figure 5). The other profilins also showed a high level of cross-reactivity to Hev b8.

## Discussion

Correct diagnosis of latex-allergy and distinction from latex-sensitization is a major task to prevent serious, potentially fatal reactions to latex, especially during diagnostic and operative procedures. In case of positive specific IgE’s against latex the intriguing challenge is to distinguish between simple sensitization and clinically relevant sensitization, i.e. latex-allergy.

Latex-allergy is mostly attributed to sensitization to Hev b5 and Hev b6. As for Hev b8 sensitized persons, mono-sensitization was seen more often than poly-sensitization to latex-specific allergens. The most serious allergic reactions with intra-operative anaphylaxis were seen in Hev b6 sensitized patients. In spite of having included healthcare workers we did not identify a single person positive to Hev b7, in contrast to Bernstein et al. [17].

The majority of latex-sensitized persons have a profilin-sensitization with mono-sensitization to Hev b8. Typically, these patients have a positive IgE CAP against latex, but are negative in SPT. In accordance with previous studies, Hev b8 mono-sensitized persons did not show latex-specific symptoms upon contact with latex-containing material in our study apart from one case [16,18,19]. The only symptoms reported in some cases were questionable or mild oral symptoms upon contact with food, e.g. kiwi or banana [8]. Thus one could speculate that in sensitization to Hev b8 the latex profilin might partly cause latex-fruit syndrome. This observation has been described in other studies [20].

The fact that two Hev b8 mono-sensitized persons were positive to bromelain (CCD) and showed symptoms upon contact with latex (contact urticaria/pruritus) raised the question whether antibodies to CCD play a major role in latex-allergy. The role of CCD is also under debate for relevant cross-reactive reactions in bee and wasp allergy or in certain unexplained allergic reactions to food.

We could show that IgE latex CAP has a high sensitivity for detection of latex-sensitized persons, whereas SPT is less sensitive but more specific in our limited patient collective for clinically relevant allergic symptoms. In accordance with other studies IgE latex CAP had high sensitivity but low specificity [4,21,22].

A limiting factor of our study is that no component-resolved diagnosis was possible for Hev b12 (lipid-transfer-protein) and for Hev b13. These might also play a role in latex-sensitized persons and be an explanation for the single case of mono-sensitized Hev b8 with symptoms upon contact [17,23]. However, in atopic patients with repeated regular exposure contact-dermatitis to latex due to late-type-sensitization as well as contact-sensitization to other substances like thiuram should be in- or excluded.

2/4 patients with discordant negative ISAC-results compared to positive ImmunoCAP test that must be considered true latex-allergic were not identified by component resolved diagnosis. The supplementation of additional recombinant latex-allergens to ISAC might enhance sensitivity of the test. However further testing concerning sensitivity of the new microarray-method compared to conventional Cap IgE is advisable.

Our study provides strong evidence that latex-allergy cannot be excluded by simply prick-testing latex. However, a positive SPT should be considered relevant as seen in our study due to high specificity, which can be explained not only by the method but also due to the fact that the prick-test-solution contains Hev b1,3,5,6 but not Hev b8.

In case of positive CAP IgE latex with negative latex SPT (or where no SPT is available), component-resolved diagnosis provides further information.

Development of a tailored biochip containing recombinant latex-allergens Hev b1,3,5,6,7,8,9,10 and bromelain might be a cost-effective variant to conventional IgE measurement in patients positive for Cap IgE latex and negative latex SPT or where latex SPT is not available.

## Abbreviations

CCD: Carbohydrate cross-reactive determinants; CRD: Component resolved diagnosis; Hev: Hevein; NRL: Natural ruber latex; SPT: Skin prick test.

## Competing interests

PSG and TH have received lecture honoraria from Thermo Fisher Scientific Phadia.

GF has received technical support by Thermo Fisher Scientific Phadia for another study.

## Authors’ contributions

SS designed the study, collected the data, performed the analysis and wrote the manuscript. GF collected the data and corrected the manuscript. PSG submitted the study to the local ethics committee, performed the analysis and corrected the manuscript. TH designed and supervised the study, performed the analysis and corrected the manuscript. All authors read and approved the final manuscript.
